# Entropy, Information, and the Updating of Probabilities

**DOI:** 10.3390/e23070895

**Published:** 2021-07-14

**Authors:** Ariel Caticha

**Affiliations:** Physics Department, University at Albany-SUNY, Albany, NY 12222, USA; acaticha@albany.edu; Tel.: +1-(518)-442-4592

**Keywords:** maximum entropy, Bayesian inference, updating probabilities

## Abstract

This paper is a review of a particular approach to the method of maximum entropy as a general framework for inference. The discussion emphasizes pragmatic elements in the derivation. An epistemic notion of information is defined in terms of its relation to the Bayesian beliefs of ideally rational agents. The method of updating from a prior to posterior probability distribution is designed through an eliminative induction process. The logarithmic relative entropy is singled out as a unique tool for updating (a) that is of universal applicability, (b) that recognizes the value of prior information, and (c) that recognizes the privileged role played by the notion of independence in science. The resulting framework—the ME method—can handle arbitrary priors and arbitrary constraints. It includes the MaxEnt and Bayes’ rules as special cases and, therefore, unifies entropic and Bayesian methods into a single general inference scheme. The ME method goes beyond the mere selection of a single posterior, and also addresses the question of how much less probable other distributions might be, which provides a direct bridge to the theories of fluctuations and large deviations.

## 1. Introduction

Inductive inference is a framework for coping with uncertainty, for reasoning with incomplete information. The framework must include a means to represent a state of partial knowledge—this is handled through the introduction of probabilities—and it must allow us to change from one state of partial knowledge to another when new information becomes available. Indeed, any inductive method that recognizes that a situation of incomplete information is in some way unfortunate—by which we mean that it constitutes a problem in need of a solution—would be severely deficient if it failed to address the question of how to proceed in those fortunate circumstances when new information becomes available. The theory of probability, if it is to be useful at all, demands a method for assigning and updating probabilities.

The challenge is to develop updating methods that are both systematic, objective and practical. When information consists of data and a likelihood function, Bayesian updating is the uniquely natural method of choice. Its foundation lies in recognizing the value of prior information: whatever was learned in the past is valuable and should not be disregarded, which amounts to requiring that beliefs ought to be revised but only to the extent required by the new data. This immediately raises a number of questions: How do we update when the information is not in the form of data? If the information is not data, what else could it possibly be? Indeed, what, after all, is “information”? On a separate line of development, the method of Maximum Gibbs–Shannon Entropy (MaxEnt) allows one to process information in the form of constraints on the allowed probability distributions. This provides a partial answer to one of our questions: in addition to data, information can also take the form of constraints. However, it immediately raises several other questions: What is the interpretation of entropy? Is there unique entropy? Are Bayesian and entropic methods mutually compatible?

The purpose of this paper is to review one particular approach to entropic updating. The presentation below, which is meant to be pedagogical and self-contained, is based on work presented in a sequence of papers [1,2,3,4,5] and in the sets of lectures [6,7,8]. As we shall see below, we adopt a pragmatic approach in which entropy is a tool designed for the specific purpose of updating probabilities.

Historically, the method of maximum relative entropy (ME) is a direct descendant of the MaxEnt method, pioneered by Jaynes [9,10]. In the MaxEnt framework, entropy is interpreted through the Shannon axioms as a measure of the amount of information that is missing in a probability distribution. This approach has its limitations. The Shannon axioms refer to probabilities of discrete variables; for continuous variables, the Shannon entropy is not defined. A more serious objection is that even if we grant that the Shannon axioms do lead to a reasonable expression for entropy, to what extent do we believe the axioms themselves? Shannon’s third axiom, the grouping property, is indeed sort of reasonable, but is it necessary? Is entropy the only consistent measure of uncertainty or of information? Indeed, there exist examples in which the Shannon entropy does not seem to reflect one’s intuitive notion of information [8,11]. One could introduce other entropies justified by different choices of axioms (e.g., [12,13,14]), but this move raises problems of its own: Which entropy should one adopt? If different systems are handled using different entropies, how does one handle composite systems?

From our perspective, the problem can be traced to the fact that neither Shannon nor Jaynes were concerned with the task of updating probabilities. Shannon’s communication theory aimed to characterize the sources of information, to measure the capacity of the communication channels, and to learn how to control the degrading effects of noise. On the other hand, Jaynes conceived MaxEnt as a method to assign probabilities on the basis of constraint information and a fixed underlying measure and not from an arbitrary prior distribution.

Considerations such as these motivated several attempts to develop ME directly as a method for updating probabilities without invoking questionable measures of information [1,5,15,16,17]. The important contribution by Shore and Johnson was the realization that one could axiomatize the updating method itself rather than the information measure. Their axioms have, however, raised criticisms [11,18,19,20] and counter-criticisms [2,6,8,21,22]. Despite the controversies, Shore and Johnson’s pioneering papers have had an enormous influence: they identified the correct goal to be achieved.

The concept of relative entropy is introduced as a tool for updating probabilities. Hereinafter, we drop the qualifier “relative”and adopt the simpler term “entropy”. The reasons for the improved nomenclature are the following: (1) The general concept should receive the general name “entropy”, while the more specialized concepts should be the ones receiving a qualifier, such as “thermodynamic” or “Clausius” entropy, and “Gibbs–Shannon” entropy. (2) All entropies are relative, even if they happen to be relative to an implicit uniform prior. Making this fact explicit has tremendous pedagogical value. (3) The practice is already in use with the concept of energy: all energies are relative too, but there is no advantage in constantly referring to a “relative energy”. Accordingly, ME will be read as “maximum entropy”; additional qualifiers are redundant.

As with all tools, entropy too is *designed* to perform a certain function, and its performance must meet certain *design criteria* or *specifications*. There is no implication that the method is “true”, or that it succeeds because it achieves some special contact with reality. Instead, the claim is that the method succeeds in the pragmatic sense that it works as designed—this is satisfactory because when properly deployed, it leads to empirically adequate models. In this approach, *entropy needs no interpretation* whether it be in terms of heat, multiplicity of states, disorder, uncertainty, or even in terms of an amount of information. Incidentally, this may explain why the search for the meaning of entropy has proved so elusive: we need not know what “entropy” means—we only need to know how to use it.

Since our topic is the updating of probabilities when confronted with new information, our starting point is to address the question, “what is information?”. In Section 2, we develop a concept of information that is both pragmatic and Bayesian. “Information” is defined in terms of its effects on the beliefs of rational agents. The design of entropy as a tool for updating is the topic of Section 3. There, we state the design specifications that define what function entropy is supposed to perform, and we derive its functional form. To streamline the presentation, some of the mathematical derivations are left to the appendices.

To conclude, we present two further developments. In Section 4, we show that Bayes’ rule can be derived as a special case of the ME method. An earlier derivation of this important result following a different line of argument was given by Williams [23] before a sufficient understanding of entropy as an updating tool had been achieved. It is not, therefore, surprising that Williams’ achievement has not received the widespread appreciation it deserves. Thus, within the ME framework, entropic and Bayesian methods are unified into a single consistent theory of inference. One advantage of this insight is that it allows a number of generalizations of Bayes’ rule [2,8]. Another is that it provides an important missing piece for the old puzzles of quantum mechanics concerning the so-called collapse of the wave function and the quantum measurement problem [24,25].

There is yet another function that the ME method must perform in order to fully qualify as a method of inductive inference. Once we have decided that the distribution of maximum entropy is to be preferred over all others, the following question arises immediately: the maximum of the entropy functional is never infinitely sharp, so are we confident that distributions that lie very close to the maximum are completely ruled out? In Section 5, the ME method is deployed to assess quantitatively the extent to which distributions with lower entropy are ruled out. The significance of this result is that it provides a direct link to the theories of fluctuations and large deviations. Concluding remarks are given in Section 6.

## 2. What Is Information?

The term “information” is used with a wide variety of different meanings [10,26,27]. There is the Shannon notion of information that is meant to measure an amount of information and is quite divorced from semantics. There is also an algorithmic notion of information that captures a notion of complexity and originates in the work of Solomonov, Kolmogorov, and Chaitin [26]; there is a related notion of entropy as a minimum description length [28]. Furthermore, in the general context of the thermodynamics of computation, it is said that “information is physical” because systems “carry” or “contain” information about their own physical state [29,30,31] (see also [32,33]).

Here, we follow a different path [3,4]. We seek an epistemic notion of information that is closer to the everyday colloquial use of the term—roughly, information is what we request when we ask a question. In a Bayesian framework, this requires an explicit account of the relation between information and the beliefs of ideally rational agents. We emphasize that our concern here is with *idealized rational agents*. Our subject is not the psychology of actual humans who often change their beliefs by processes that are neither fully rational nor fully conscious. We adopt a Bayesian interpretation of probability as a degree of credibility: the degree to which we *ought* to believe that a proposition is true if only we were ideally rational. For a discussion of a decision theory that might be relevant to the economics and psychology of partially rational agents see [34,35,36]. An entropic framework for modelling economies that bypasses all issues of bounded rationality is described in [37].

It is implicit in the recognition that most of our beliefs are held on the basis of incomplete information that not all probability assignments are equally good; some beliefs are preferable to others in the very pragmatic sense that they enhance our chances to successfully navigate this world. Thus, a theory of probability demands a theory of updating probabilities in order to improve our beliefs.

We are now ready to address the question: What, after all, is “information”? The answer is pragmatic. *Information is what information does.* Information is defined by its effects: (a) it restricts our options as to what we are honestly and rationally allowed to believe; and (b) it induces us to update from prior beliefs to posterior beliefs. This, I propose, is a defining characteristic of information:

*Information is that which induces a change from one state of rational belief to another.*

One aspect of this notion is that for a rational agent, the identification of what constitutes information—as opposed to mere noise—already involves a judgment, an evaluation. Another aspect is that the notion that information is directly related to changing our minds does not involve any reference to *amounts* of information, but it nevertheless allows precise quantitative calculations. Indeed, constraints on the acceptable posterior probabilities are precisely the kind of information that the method of maximum entropy is designed to handle. In short,

*Information constrains probability distributions. The constraints are the information.*

To the extent that the probabilities are Bayesian, this definition captures the Bayesian notion that information is directly related to changing our minds, that it is the driving force behind the process of learning. It also incorporates an important feature of rationality: being rational means accepting that “not everything goes”, and that our beliefs must be constrained in very specific ways. However, the indiscriminate acceptance of any arbitrary constraint does not qualify as rational behavior. To be rational, an agent must exercise some judgment before accepting a particular piece of information as a reliable basis for the revision of its beliefs, which raises questions about what judgments might be considered sound. Furthermore, there is no implication that the information must *be true*; only that we *accept it as true*. False information is information too, at least to the extent that we are prepared to accept it and allow it to affect our beliefs.

The paramount virtue of the definition above is that it is useful; it allows precise quantitative calculations. The constraints that constitute information can take a wide variety of forms. They can be expressed in terms of expected values, they can specify the functional form of a distribution, or be imposed through various geometrical relations. Examples are given in Section 5 and in [38].

Concerning the act of updating, it may be worthwhile to point out an analogy with dynamics. In Newtonian mechanics, the state of motion of a system is described in terms of momentum, and the change from one state to another is said to be “caused” by an applied force or impulse. Bayesian inference is analogous in that a state of belief is described in terms of probabilities, and the change from one state to another is “caused” by information. Just as a force is that which induces a change from one state of motion to another, so *information is that which induces a change from one state of belief to another*. Updating is a form of dynamics. In [39], the analogy is taken seriously: the logic is reversed and quantum mechanics is derived as an example of the entropic updating of probabilities.

## 3. The Pragmatic Design of Entropic Inference

Once we have decided, as a result of the confrontation of new information with old beliefs, that our beliefs require revision, the problem becomes one of deciding how precisely this ought to be done. First, we identify some general features of the kind of belief revision that one might count as rational. Then, we design a method—a systematic procedure—that implements those features. To the extent that the method performs as desired, we can claim success. The point is not that success derives from our method having achieved some intimate connection to the inner wheels of reality; success simply means that the method seems to be working.

The one obvious requirement is that the updated probabilities ought to agree with the newly acquired information. Unfortunately, this requirement, while necessary, is not sufficiently restrictive: we can update in many ways that preserve both internal consistency and consistency with the new information. Additional criteria are needed. What rules would an ideally rational agent choose?

### 3.1. General Criteria

The rules are motivated by the same pragmatic criteria that motivate the design of probability theory itself [8]—universality, consistency, and practical utility. However, this is admittedly too vague; we must be very specific about the precise way in which the criteria are implemented.

#### 3.1.1. Universality

In principle, different systems and different situations could require different problem-specific induction methods. However, in order to be useful in practice, the method we seek must be of *universal* applicability. Otherwise, it would fail us when most needed, for we would not know which method to choose when not much is known about the system. To put in different words, what we want to design is a general-purpose method that captures what all the other problem-specific methods might have in common. The idea is that the peculiarities of a particular problem will be captured by the specific constraints that describe the information that is relevant to the problem at hand.

The analogy with mechanics can be found here as well. The possibility of a science of mechanics hinges on identifying a law of motion of universal applicability (e.g., the Schrödinger equation), while the specifics of each system are introduced through initial conditions and the choice of potentials or forces. Here, we shall design an entropy of universal applicability, while the specifics of each problem are introduced through prior probabilities and the choice of constraints.

#### 3.1.2. Parsimony

To specify the updating, we adopt a very conservative criterion that recognizes the value of information: what has been laboriously learned in the past is valuable and should not be disregarded unless rendered obsolete by new information. The only aspects of one’s beliefs that should be updated are those for which new evidence has been supplied. Thus, we adopt the following.

**Principle of Minimal Updating (PMU)**: *Beliefs should be updated only to the minimal extent required by the new information.*

The special case of updating in the absence of new information deserves a comment. The PMU states that when there is no new information, ideally, rational agents should not change their minds. In fact, it is difficult to imagine any notion of rationality that would allow the possibility of changing one’s mind for no apparent reason.

Minimal updating offers yet another pragmatic advantage. As we shall see below, rather than identifying what features of a distribution are singled out for updating and then specifying the detailed nature of the update, we will adopt design criteria that stipulate what is not to be updated. The practical advantage of this approach is that it enhances objectivity—there are many ways to change something but only one way to keep it the same. The analogy with mechanics can be pursued even further: if updating is a form of dynamics, then minimal updating is the analogue of inertia. Rationality and objectivity demand a considerable amount of inertia.

#### 3.1.3. Independence

The next general requirement turns out to be crucially important because without it, the very possibility of scientific theories would be compromised. The point is that every scientific model, whatever the topic, if it is to be useful at all, must assume that all relevant variables have been taken into account and that whatever was left out—the rest of the universe—should not matter. To put it another way, in order to do scientific work, we must be able to understand parts of the universe without having to understand the universe as a whole. Granted, a pragmatic understanding need not be complete and exact; it must be merely adequate for our purposes.

The assumption, then, is that it is possible to focus our attention on a suitably chosen system of interest and neglect the rest of the universe because the system and the rest of the universe are “sufficiently independent”. Thus, in any form of science, the notion of statistical independence must play a central and privileged role. This idea—that some things can be neglected and that not everything matters—is implemented by imposing a criterion that tells us how to handle independent systems. The chosen criterion is quite natural: *whenever two systems are a priori believed to be independent and we receive information about just one, it should not matter if the other is included in the analysis or not.* This is an example of the PMU in action; it amounts to requiring that independence to be preserved unless information about correlations is explicitly introduced.

Again, we emphasize that none of these criteria are imposed by nature. They are desirable for pragmatic reasons; they are imposed by design.

### 3.2. Entropy as a Tool for Updating Probabilities

Consider a set of propositions {x} about which we are uncertain. The proposition *x* can be discrete or continuous, in one or in several dimensions. It could, for example, represent the microstate of a physical system, a point in phase space, or an appropriate set of quantum numbers. The uncertainty about *x* is described by a probability distribution q(x). The goal is to update from the prior distribution q(x) to a posterior distribution p(x) when new information—by which we mean a set of constraints—becomes available. The question is, which distribution among all those that satisfy the constraints should we select?

Our goal is to design a method that allows a systematic search for the preferred posterior distribution. The central idea, first proposed by Skilling [16], is disarmingly simple: to select the posterior, first rank all candidate distributions in increasing order of “preference” and then pick the distribution that ranks the highest. Irrespective of what it is that makes one distribution “preferable” over another (we will get to that soon enough), it is clear that any such ranking must be transitive: if distribution $p_{1}$ is preferred over distribution $p_{2}$, and $p_{2}$ is preferred over $p_{3}$, then $p_{1}$ is preferred over $p_{3}$. Transitive rankings are implemented by assigning to each *p* a real number S[p], which is called the entropy of *p* in such a way that if $p_{1}$ is preferred over $p_{2}$, then $S\left[p_{1}\right]>S\left[p_{2}\right]$. The selected distribution (one or possibly many, for *there may be several equally preferred distributions*) is that which maximizes the entropy functional.

The importance of Skilling’s strategy of ranking distributions cannot be overestimated: it answers the questions “why entropy?” and “why a maximum?”. The strategy implies that the updating method will take the form of a variational principle—the method of maximum entropy (ME)—involving a certain functional that maps distributions to real numbers. These features are not imposed by nature; they are all imposed by design. They are dictated by the function that the ME method is supposed to perform. (Thus, it makes no sense to seek a generalization in which entropy is a complex number or a vector; such generalized entropies would just not perform the desired function.)

Next, we specify the ranking scheme, that is, we choose a specific functional form for the entropy S[p]. Note that the purpose of the method is to update *from priors to posteriors* so the ranking scheme must depend on the particular prior *q* and therefore, the entropy *S* must be a functional of both *p* and *q*. The entropy S[p,q] describes a ranking of the distributions *p relative* to the given prior *q*. S[p,q] is the entropy of *p relative* to *q*, and accordingly, S[p,q] is commonly called *relative entropy*. This is appropriate and sometimes we will follow this practice. However, since all entropies are relative, even when relative to a uniform distribution, the qualifier “relative” is redundant and can be dropped.

The functional S[p,q] is designed by a process of elimination—this is a process of *eliminative induction*. First, we state the desired design criteria; this is the crucial step that defines what makes one distribution preferable over another. Candidate functionals that fail to satisfy the criteria are discarded—hence, the qualifier “eliminative”. As we shall see, the criteria adopted below are so constraining that there is a single entropy functional S[p,q] that survives the process of elimination.

This approach has a number of virtues. First, to the extent that the design criteria are universally desirable, the single surviving entropy functional will also be of universal applicability. Second, the reason why alternative entropy candidates are eliminated is quite explicit—at least one of the design criteria is violated. Thus, *the justification behind the single surviving entropy is not that it leads to demonstrably correct inferences, but rather, that all other candidates demonstrably fail to perform as desired.*

### 3.3. Specific Design Criteria

Consider a lattice of propositions generated by a set X of atomic propositions that are mutually exclusive and exhaustive and are labeled by a discrete index i=1,2,…,n. The extension to infinite sets and to continuous labels turns out to be straightforward. The index *i* might, for example, label the microstates of a physical system but, since the argument below is supposed to be of general validity, we shall not assume that the labels themselves carry any particular significance. We can always permute labels; this should have no effect on the updating of probabilities.

We adopt design criteria that reflect the structure of the lattice of propositions—the propositions are related to each other by disjunctions (or) and conjunctions (and) and the consistency of the web of beliefs is implemented through the sum and product rules of the probability theory. Our criteria refer to the two extreme situations of propositions that are mutually exclusive and of propositions that are mutually independent. At one end, we deal with the probabilities of propositions that are highly correlated (if one proposition is true, the other is false and vice versa); at the other end, we deal with the probabilities of propositions that are totally uncorrelated (the truth or falsity of one proposition has no effect on the truth or falsity of the other). One extreme is described by the simplified sum rule, p(i∨j)=p(i)+p(j), and the other extreme by the simplified product rule, p(i∧j)=p(i)p(j). (For an alternative approach to the foundations of inference that exploits the various symmetries of the lattice of propositions see [40,41].

The two design criteria and their consequences for the functional form of entropy are given below. Detailed proofs are deferred to the appendices.

#### 3.3.1. Mutually Exclusive Subdomains

**DC1**: *Probabilities that are conditioned on one subdomain are not affected by information about other non-overlapping subdomains.*

Consider a subdomain D⊂X composed of atomic propositions i∈D and suppose the information to be processed refers to some other subdomain $D^{\prime }\subset X$ that does not overlap with D, $D\cap D^{\prime }=\emptyset$. In the absence of any new information about D, the PMU demands, we do not change our minds about probabilities that are conditional on D. Thus, we design the inference method so that q(i|D), the prior probability of *i* conditioned on i∈D, is not updated. Thus, the selected conditional posterior is

(1) P(i|D)=q(i|D).

We adopt the following notation: priors are denoted by *q*, candidate posteriors by lower case *p*, and the selected posterior by upper case *P*. We shall write either p(i) or $p_{i}$. Furthermore, we adopt the notation, standard in physics where the probabilities of *x* and θ are written p(x) and p(θ) but there is no implication that *p* refers to the same mathematical function.

We emphasize that the point is not that we make the unwarranted assumption that keeping q(i|D) unchanged is guaranteed to lead to correct inferences. It need not; induction is risky. The point is, rather, that in the absence of any evidence to the contrary, there is no reason to change our minds and the prior information takes priority.

**The consequence of DC1** is that non-overlapping domains of *i* contribute additively to the entropy,

(2) $$S\left(p,q\right)=\sum_{i}\;F\left(p_{i},q_{i}\right)\;,$$

where *F* is some unknown function of two arguments. The proof is given in Appendix A.

**Comment 1:** It is essential that DC1 refers to *conditional* probabilities—local information about a domain $D^{\prime }$ can (via normalization) have a non-local effect on the probability of another domain D.

**Comment 2:** An important special case is the “update” from a prior q(i) to a posterior P(i) in a situation in which no new information is available. The criterion DC1 applied to a situation where the subdomain D covers the whole space of is, D=X, requires that *in the absence of any new information, the prior conditional probabilities are not to be updated:* P(i|X)=q(i|X)*or*P(i)=q(i).

**Comment 3:** The criterion DC1 implies Bayesian conditionalization as a special case. Indeed, if the information is given through the constraint $p(\tilde{D})=0$, where $\tilde{D}$ is the complement of D, then P(i|D)=q(i|D), which is referred to as Bayesian conditionalization. More explicitly, if θ is the variable to be inferred on the basis of prior information about a likelihood function q(i|θ) and observed data $i^{\prime }$, then the update from the prior *q* to the posterior *P*, is

(3) q(i,θ)=q(i)q(θ|i)→P(i,θ)=P(i)P(θ|i),

consists of updating $q\left(i\right)\to P\left(i\right)=\delta_{ii^{\prime }}$ to agree with the new information and invoking the PMU so that $P\left(\theta |i^{\prime }\right)=q\left(\theta |i^{\prime }\right)$ remains unchanged. Therefore,

(4) $$P\left(i,\theta \right)=\delta_{ii^{\prime }}q\left(\theta |i^{\prime }\right)\;\mathrm{so}\;\mathrm{that}\;P\left(\theta \right)=q\left(\theta |i^{\prime }\right)=q\left(\theta \right)\frac{q(i^{\prime }|\theta )}{q(i^{\prime })}\;,$$

which is Bayes’ rule. Thus, *entropic inference is designed to include Bayesian inference as a special case*. Note, however, that imposing DC1 is not identical to imposing Bayesian conditionalization: DC1 is not restricted to information in the form of absolute certainties, such as p(D)=1.

**Comment 4:** If the label *i* is turned into a continuous variable *x*, the criterion DC1 requires that information that refers to points infinitely close but just outside the domain D will have no influence on probabilities conditional on D. This may seem surprising, as it may lead to updated probability distributions that are discontinuous, but it is not a problem. In situations where we have explicit reasons to believe that conditions of continuity or differentiability hold, then such conditions should be imposed explicitly. The inference process should not be expected to discover and replicate information with which it was not supplied.

#### 3.3.2. Subsystem Independence

**DC2**: *When two systems are a priori believed to be independent and the information we receive about one of them makes no reference to the other, then it should not matter whether the latter is included in the analysis of the former or not.*

Consider a system of propositions labeled by a composite index, $i=\left(i_{1},i_{2}\right)\in X=X_{1}\times X_{2}$. For example, $\left\{i_{1}\right\}=X_{1}$ and $\left\{i_{2}\right\}=X_{2}$ might describe the microstates of two separate physical systems. Assume that all prior evidence led us to believe the two subsystems are independent, that is, any two propositions $i_{1}\in X_{1}$ and $i_{2}\in X_{2}$ are believed to be independent. This belief is reflected in the prior distribution: if the individual subsystem priors $q_{1}\left(i_{1}\right)$ and $q_{2}\left(i_{2}\right)$, then the prior for the whole system is $q_{1}\left(i_{1}\right)q_{2}\left(i_{2}\right)$. Next, suppose that new information is acquired such that $q_{1}\left(i_{1}\right)$ would by itself be updated to $P_{1}\left(i_{1}\right)$, and that $q_{2}\left(i_{2}\right)$ would by itself be updated to $P_{2}\left(i_{2}\right)$. DC2 requires that S[p,q] be such that the joint prior $q_{1}\left(i_{1}\right)q_{2}\left(i_{2}\right)$ updates to the product $P_{1}\left(i_{1}\right)P_{2}\left(i_{2}\right)$ so that inferences about one subsystem do not affect inferences about the other.

**The consequence of DC2** is to fully determine the unknown function *F* in (2) so that probability distributions p(i) should be ranked relative to the prior q(i) according to the relative entropy,

(5) $$S\left[p,q\right]=-\sum_{i}\;p\left(i\right)\log\frac{p(i)}{q(i)}.$$

**Comment 1:** We emphasize that the point is not that when we have no evidence for correlations, we draw the firm conclusion that the systems must necessarily be independent. Induction involves risk; the systems might, in actual fact, be correlated through some unknown interaction potential. The point is rather that if the joint prior reflected independence and the new evidence is silent on the matter of correlations, then the evidence we actually have—namely, the prior—takes precedence, and there is no reason to change our minds. As before, the PMU requires that a feature of the probability distribution—in this case, independence—will not be updated unless the evidence requires it.

**Comment 2:** We also emphasize that DC2 *is not a consistency requirement*. The argument we deploy is *not* that both the prior *and* the new information tell us the systems are independent in which case consistency requires that it should not matter whether the systems are treated jointly or separately. DC2 refers to a situation where the new information does not say whether the systems are independent or not. Rather, the updating is being *designed*—through the PMU—so that the independence reflected in the prior is maintained in the posterior by default.

**Comment 3:** The generalization to continuous variables x∈X is approached as a Riemann limit from the discrete case. A continuous probability density p(x) or q(x) can be approximated by the discrete distributions. Divide the region of interest X into a large number *N* of small cells. The probabilities of each cell are as follows:

(6) $$p_{i}=p\left(x_{i}\right)\Delta x_{i}\;\mathrm{and}\;q_{i}=q\left(x_{i}\right)\Delta x_{i}\;,$$

where $\Delta x_{i}$ is an appropriately small interval. The discrete entropy of $p_{i}$ relative to $q_{i}$ is as follows:

(7) $$S_{N}=-\sum_{i=1}^{N}\Delta x_{i}\;p\left(x_{i}\right)\;\log\left[\frac{p\left(x_{i}\right)\Delta x_{i}}{q\left(x_{i}\right)\Delta x_{i}}\right]\;\;,$$

and in the limit as N→∞ and $\Delta x_{i}\to 0$ we get the Riemann integral

(8) $$S\left[p,q\right]=-\int dx\;p\left(x\right)\;\log\;\left[\frac{p(x)}{q(x)}\right]\;.$$

(To simplify the notation, we include multi-dimensional integrals by writing $d^{n}x=dx$.) It is easy to check that the ranking of distributions induced by S[p,q] is invariant under coordinate transformations. The insight that coordinate invariance could be derived as a consequence of the requirement of subsystem independence first appeared in [5].

### 3.4. The ME Method

We can now summarize the overall conclusion.

**The ME method**: *The goal is to update from a prior distribution q to a posterior distribution when there is new information in the form of constraints*C*that specify a family*{p}*of candidate posteriors. The preferred posterior P is that which maximizes the relative entropy,*

(9) $$S\left[p,q\right]=-\sum_{i}\;p_{i}\log\frac{p_{i}}{q_{i}}\;\mathrm{or}\;S\left[p,q\right]=-\int dx\;p\left(x\right)\;\log\;\left[\frac{p(x)}{q(x)}\right]\;,$$

*within the family*{p}*specified by the constraints*C.

This extends the method of maximum entropy beyond its original purpose as a rule to assign probabilities from a given underlying measure (MaxEnt) to a method for updating probabilities from any arbitrary prior (ME). Furthermore, the logic behind the updating procedure does not rely on any particular meaning assigned to the entropy whether in terms of information, or heat, or disorder. Entropy is merely a tool for inductive inference. *No interpretation for* S[p,q] *is given and none is needed.*

The derivation above has singled out *a unique* S[p,q] *to be used in inductive inference*. Other “entropies” (such as the one-parameter families of entropies proposed in [12,13,14] might turn out to be useful for other purposes—perhaps as measures of some kind of “information”, as measures of discrimination or distinguishability among distributions, of ecological diversity, or for some altogether different function—but they are unsatisfactory for the purpose of updating because they fail to perform the functions stipulated by the design criteria DC1 and DC2. They induce correlations that are unwarranted by the information in the priors or the constraints.

## 4. Bayes’ Rule as a Special Case of ME

Back in Section 3.3.1, we saw that ME is designed to include Bayes’ rule as a special case. Here, we wish to verify this explicitly [2]. The goal is to update our beliefs about θ∈Θ (θ represents one or many parameters) on the basis of three pieces of information: (1) the prior information codified into a prior distribution q(θ); (2) the new information conveyed by data x∈X (obtained in one or many experiments); and (3) the known relation between θ and *x* given by a model defined by the sampling distribution or likelihood, q(x|θ). The updating will result in replacing the *prior* probability distribution q(θ) by a *posterior* distribution P(θ) that applies after the data information has been processed.

The crucial element that will allow the Bayes’ rule to be smoothly integrated into the ME scheme is the realization that before the data are collected, not only do we not know θ, but we do not know *x* either. Thus, the relevant space for inference is not the space Θ but the product space Θ×X, and the relevant joint *prior* is q(x,θ)=q(θ)q(x|θ). Let us emphasize two points: first, the likelihood function is an integral part of the *prior* distribution; second, the prior information about how *x* is related to θ is contained in the *functional form* of the distribution q(x|θ) and not in the numerical values of the arguments *x* and θ, which, at this point, are still unknown.

Next, data are collected and the observed values turn out to be $x^{\prime }$. We must update to a posterior that lies within the family of distributions p(x,θ) that reflect the fact that the previously unknown *x* is now known to be $x^{\prime }$, that is,

(10) $$p\left(x\right)=\int d\theta \;p\left(\theta ,x\right)=\delta \left(x-x^{\prime }\right)\;.$$

The information in this data constrains but is not sufficient to fully determine the joint distribution,

(11) $$p\left(x,\theta \right)=p\left(x\right)p\left(\theta |x\right)=\delta \left(x-x^{\prime }\right)p\left(\theta |x^{\prime }\right)\;.$$

Any choice of $p(\theta |x^{\prime })$ is, in principle, possible. So far, the formulation of the problem parallels Section 3.3.1 exactly. We are, after all, solving the same problem. The next step is to apply the ME method.

According to the ME method, the selected joint posterior P(x,θ) is that which maximizes the entropy,

(12) $$S\left[p,q\right]=-\int dxd\theta \;p\left(x,\theta \right)\log\frac{p(x,\theta )}{q(x,\theta )}\;,\;$$

subject to the data constraints. Note that Equation (10) represents an *infinite* number of constraints on the family p(x,θ): there is one constraint and one Lagrange multiplier λ(x) for each value of *x*. Maximizing *S*, (12), subject to (10) and normalization,

(13) $$\begin{array}{cc} \delta \left\{S+\alpha \left[\int dxd\theta \;p(x,\theta )-1\right]+\int dx\;\lambda \left(x\right)\left[\int d\theta \;p\left(x,\theta \right)-\delta \left(x-x^{\prime }\right)\right]\right\} & =0\;, \end{array}$$

yields the joint posterior

(14) $$P\left(x,\theta \right)=q\left(x,\theta \right)\;\frac{e^{\lambda (x)}}{Z}\;,$$

where *Z* is a normalization constant, and the multiplier λ(x) is determined from (10) as follows:

(15) $$\int d\theta \;q\left(x,\theta \right)\frac{\;e^{\lambda (x)}}{Z}=q\left(x\right)\frac{\;e^{\lambda (x)}}{Z}=\delta \left(x-x^{\prime }\right)\;,$$

so that the joint posterior is

(16) $$P\left(x,\theta \right)=q\left(x,\theta \right)\frac{\;\delta (x-x^{\prime })}{q(x)}=\delta \left(x-x^{\prime }\right)q\left(\theta |x\right)\;.$$

The corresponding marginal posterior probability P(θ) is

(17) $$P\left(\theta \right)=\int dx\;P\left(\theta ,x\right)=q\left(\theta |x^{\prime }\right)=q\left(\theta \right)\frac{q(x^{\prime }|\theta )}{q(x^{\prime })}\;,$$

which is Bayes’ rule. Thus, Bayes’ rule is derivable from, and therefore consistent with, the ME method.

To summarize, the prior q(x,θ)=q(x)q(θ|x) is updated to the posterior P(x,θ)=P(x)P(θ|x), where $P\left(x\right)=\delta \left(x-x^{\prime }\right)$ is fixed by the observed data while $P\left(\theta |x^{\prime }\right)=q\left(\theta |x^{\prime }\right)$ remains unchanged. Note that in accordance with the PMU philosophy that drives the ME method, *one only updates those aspects of one’s beliefs for which corrective new evidence has been supplied*. In [2,8,42], further examples are given that show how ME allows generalizations of Bayes’ rule to situations where the data itself are uncertain, there is information about moments of *x* or moments of θ, or even in situations where the likelihood function is unknown. In conclusion, the ME method of maximum entropy can fully reproduce and then go beyond the results obtained by the standard Bayesian methods.

## 5. Deviations from Maximum Entropy

The basic ME problem is to update from a prior q(x) given information specified by certain constraints. The constraints specify a family of candidate distributions as follows:

(18) $$p_{\theta }\left(x\right)=p\left(x|\theta \right)$$

which can be conveniently labeled with a finite number of parameters $\theta^{a}$, a=1…n. (The generalization to an infinite number of parameters poses technical but not insurmountable difficulties.) Thus, the parameters θ are coordinates on the statistical manifold specified by the constraints. The distributions in this manifold are ranked according to their entropy,

(19) $$S\left[p_{\theta },q\right]=-\int dx\;p\left(x|\theta \right)\;\log\frac{p(x|\theta )}{q(x)}=S\left(\theta \right)\;,$$

and the selected posterior is the distribution $p(x|\theta_{0})$ that maximizes the entropy S(θ). (The notation indicates that $S[p_{\theta },q]$ is a functional of $p_{\theta }$ while S(θ) is a function of θ.)

The question we now address concerns the extent to which $p(x|\theta_{0})$ should be preferred over other distributions with lower entropy or, to put it differently, to what extent is it rational to believe that the selected value ought to be the entropy maximum $\theta_{0}$ rather than any other value θ [1]? This is a question about the probability p(θ) of various values of θ. The original problem which led us to design the maximum entropy method was to assign a probability to the quantity *x*; we now see that the full problem is to assign probabilities to both *x* and θ. We are concerned not just with p(x), but rather with the joint distributions which we denote as π(x,θ); the universe of discourse has been expanded from X (the space of *x*s) to the product space X×Θ (Θ is the space of parameters θ).

To determine the joint distribution π(x,θ), we make use of essentially the only (universal) method at our disposal—the ME method itself—but this requires that we address the standard two preliminary questions: First, what is the prior distribution? What do we know about *x* and θ before we receive information about the constraints? Second, what is the new information that constrains the allowed joint distributions π(x,θ)?

This first question is the more subtle one: when we know absolutely nothing about the θs, we know neither their physical meaning nor whether there is any relation to the *x*s. A joint prior that reflects this lack of correlations is a product, q(x,θ)=q(x)q(θ). We will assume that the prior q(x) is known—it is the same prior we had used when we updated from q(x) to $p(x|\theta_{0})$ using (19).

However, we are not totally ignorant about the θs: we know that they label distributions π(x|θ) on some as yet unspecified statistical manifold Θ. Then there exists a natural measure of distance in the space Θ. It is given by the information metric $d\ell^{2}=g_{ab}d\theta^{a}d\theta^{b}$ [8,43], where

(20) $$g_{ab}=\int dx\;p\left(x|\theta \right)\frac{\partial \log p(x|\theta )}{\partial \theta^{a}}\frac{\partial \log p(x|\theta )}{\partial \theta^{b}}\;,$$

and the corresponding volume elements are given by $g^{1/2}\left(\theta \right)d^{n}\theta$, where g(θ) is the determinant of the metric. The uniform prior for θ, which assigns equal probabilities to equal volumes, is proportional to $g^{1/2}\left(\theta \right)$, and therefore we choose $q\left(\theta \right)=g^{1/2}\left(\theta \right)$. Therefore, the joint prior is $q\left(x,\theta \right)=q\left(x\right)g^{1/2}\left(\theta \right)$.

Next, we tackle the second question: what are the constraints on the allowed joint distributions π(x,θ)? Consider the space of all joint distributions. To each choice of the functional form of π(x|θ) (for example, whether we talk about Gaussians, Boltzmann–Gibbs distributions, or something else), there corresponds a different subspace defined by distributions of the form π(x,θ)=π(θ)π(x|θ). The crucial constraint is that which specifies the subspace by imposing that π(x|θ) takes the particular functional form given by the constraint (18), π(x|θ)=p(x|θ). This defines the meaning to the θs and also fixes the prior $g^{1/2}\left(\theta \right)$ on the relevant subspace.

The preferred joint distribution, P(x,θ)=P(θ)p(x|θ), is the distribution, π(x,θ)=π(θ)p(x|θ), that maximizes the joint entropy,

(21) $$\begin{array}{cc} S[\pi ,q] & =-\int dx\;d\theta \;\pi \left(\theta \right)p\left(x|\theta \right)\;\log\frac{\pi (\theta )p(x|\theta )}{g^{1/2}\left(\theta \right)q\left(x\right)}\\ \; & =-\int \;d\theta \;\pi \left(\theta \right)\log\frac{\pi (\theta )}{g^{1/2}\left(\theta \right)}+\int d\theta \;\pi \left(\theta \right)S\left(\theta \right)\;, \end{array}$$

where S(θ) is given in (19). Varying (21) with respect to π(θ) with ∫dθπ(θ)=1 and p(x|θ) fixed yields the posterior probability that the value of θ lies within the small volume $g^{1/2}\left(\theta \right)d^{n}\theta$,

(22) $$P\left(\theta \right)d^{n}\theta =\frac{1}{\zeta }\;\;e^{S(\theta )}g^{1/2}\left(\theta \right)d^{n}\theta \;\mathrm{with}\;\zeta =\int d^{n}\theta \;g^{1/2}\left(\theta \right)\;e^{S(\theta )}.$$

Equation (22) is the result we seek. It tells us that, as expected, the preferred value of θ is the value $\theta_{0}$ that maximizes the entropy S(θ), Equation (19), because this maximizes the scalar density expS(θ). However, it also tells us the degree to which values of θ away from the maximum are ruled out. (Note that the density expS(θ) is a scalar function and the presence of the Jacobian factor $g^{1/2}\left(\theta \right)$ makes Equation (22) manifestly invariant under changes of the coordinates θ in the space Θ.)

This discussion allows us to refine our understanding of the ME method. ME is not an all-or-nothing recommendation to pick the single distribution that maximizes entropy and reject all others. The ME method is more nuanced: in principle, all distributions within the constraint manifold ought to be included in the analysis; they contribute in proportion to the exponential of their entropy and this turns out to be significant in situations where the entropy maximum is not particularly sharp.

Going back to the original problem of updating from the prior q(x), given information that specifies the manifold {p(x|θ)}, the preferred update within the family {p(x|θ)} is $p(x|\theta_{0})$, but to the extent that other values of θ are not totally ruled out, a better update is obtained marginalizing the joint posterior P(x,θ)=P(θ)p(x|θ) over θ,

(23) $$P\left(x\right)=\int d^{n}\theta \;P\left(\theta \right)p\left(x|\theta \right)=\int d^{n}\theta \;g^{1/2}\left(\theta \right)\frac{\;e^{S(\theta )}}{\zeta }p\left(x|\theta \right)\;.$$

In situations where the entropy maximum at $\theta_{0}$ is very sharp, we recover the old result,

(24) $$P\left(x\right)\approx p\left(x|\theta_{0}\right)\;.$$

When the entropy maximum is not very sharp a more honest update is Equation (23), which, incidentally, is a form of superstatistics.

One of the limitations of the standard MaxEnt method is that it selects a single “posterior” $p(x|\theta_{0})$ and strictly rules out all other distributions. The result (22) overcomes this limitation and finds many applications. For example, it extends the Einstein theory of thermodynamic fluctuations beyond the regime of small fluctuations; it provides a bridge to the theory of large deviations; and, suitably adapted for Bayesian data analysis, it leads to the notion of entropic priors [44].

## 6. Discussion

Consistency with the law of large numbers.

Entropic methods of inference are of general applicability but there exist special situations—for example, those involving large numbers of independent subsystems—where inferences can be made by purely probabilistic methods without ever invoking the concept of entropy. In such cases, one can check (see, for example, [6,45]) that the two methods of calculation are consistent with each other. It is significant, however, that alternative entropies, such as those proposed in [12,13,14], do not pass this test [46,47], which rules them out as tools for updating. Some probability distributions obtained by maximizing the alternative entropies have, however, turned out to be physically relevant. It is, therefore, noteworthy that those successful distributions can also be derived through a more standard application of MaxEnt or ME, as advocated in this review [8,48,49,50,51]. In other words, what is being ruled out are not the distributions themselves, but the alternative entropies from which they were inferred.

On priors.

Choosing the prior density q(x) can be tricky. Sometimes, symmetry considerations can be useful but otherwise, there is no fixed set of rules to translate information into a probability distribution except, of course, for Bayes’ rule and the ME method themselves.

What if the prior q(x) vanishes for some values of *x*? S[p,q] can be infinitely negative when q(x) vanishes within some region D. This means that the ME method confers an infinite preference on those distributions p(x) that vanish whenever q(x) does. One must emphasize that this is as it should be. A similar situation also arises in the context of Bayes’ theorem, where assigning a vanishing prior represents a tremendously serious commitment because no amount of data to the contrary would allow us to revise it. In both ME and Bayes updating, we should recognize the implications of assigning a vanishing prior. Assigning a very low but non-zero prior represents a safer and possibly less prejudiced representation of one’s prior beliefs.

Commuting and non-commuting constraints.

The ME method allows one to process information in the form of constraints. When we are confronted with several constraints, we must be particularly cautious. Should they be processed simultaneously or sequentially? And, if the latter, in what order? The answer depends on the problem at hand [42].

We refer to constraints as *commuting* when it makes no difference whether they are handled simultaneously or sequentially. The most common example is that of Bayesian updating on the basis of data collected in several independent experiments. In this case, the order in which the observed data $x^{\prime }=\left\{x_{1}^{\prime },x_{2}^{\prime },\ldots \right\}$ are processed does not matter for the purpose of inferring θ. In general, however, constraints need not commute and when this is the case, the order in which they are processed is critical.

To decide whether constraints are to be handled sequentially or simultaneously, one must be clear about how the ME method handles constraints. The ME machinery interprets a constraint in a very mechanical way: all distributions satisfying the constraint are, in principle, allowed, while all distributions violating it are ruled out. Therefore, sequential updating is appropriate when old constraints become obsolete and are superseded by new information, while simultaneous updating is appropriate when old constraints remain valid. The two cases refer to different states of information, and therefore, it is to be expected that they will result in different inferences. These comments are meant to underscore the importance of understanding what information is and how it is processed by the ME method; failure to do so will lead to errors that do not reflect a shortcoming of the ME method but rather a misapplication of it.

Pitfalls?

Entropy is a tool for reasoning and—as with all tools for reasoning or otherwise—it can be misused, leading to unsatisfactory results [52]. Should that happen, the inevitable questions are “what went wrong?” and “how do we fix it?” It helps to first ask what components of the analysis can be trusted so that the possible mistakes can be looked for elsewhere. The answers proposed by the ME method are radically conservative: problems always arise through a wrong choices of variables, priors, or constraints. Indeed, one should not blame the entropic method for not having discovered and taken into account relevant information that was not explicitly introduced into the analysis. Indeed, just as one would be very reticent about questioning the basic rules of arithmetic, or the basic rules of calculus, one should not question the basic sum and product rules of the probability calculus and, taking this one step farther, one should not question the applicability of entropy as the updating tool. The adoption of this conservative approach leads us to reject alternative entropies and quantum probabilities. Fortunately, those constructs are not actually needed—as mentioned above, those Tsallis distributions that have turned out be useful can be derived with standard entropic methods [8,48,49,50,51], and quantum mechanics can be handled within standard probability theory without invoking exotic probabilities [39,53].

## Data Availability

Not applicable.

## Appendix A. DC1—Mutually Exclusive Subdomains

In these appendices, we establish the consequences of the two criteria DC1 and DC2, leading to the final result: Equation (9). The details of the proofs are important not just because they lead to our final conclusions, but also because the translation of the verbal statement of the criteria into precise mathematical form is a crucial part of unambiguously specifying what the criteria actually say.

First, we prove that criterion DC1 leads to the expression Equation (2) for S[p,q]. Consider the case of a discrete variable, $p_{i}$ with i=1…n, so that $S\left[p,q\right]=S\left(p_{1}\ldots p_{n},q_{1}\ldots q_{n}\right)$. Suppose the space of states X is partitioned into two non-overlapping domains D and $\tilde{D}$ with $D\cup \tilde{D}=X$, and that the information to be processed is in the form of a constraint that refers to the domain $\tilde{D}$,

(A1) $$\sum_{j\in \tilde{D}}a_{j}p_{j}=A\;.$$

DC1 states that the constraint on $\tilde{D}$ does not have an influence on the *conditional* probabilities $p_{i|D}$. It may, however, influence the probabilities $p_{i}$ within D through an overall multiplicative factor. To deal with this complication, consider then a special case where the overall probabilities of D and $\tilde{D}$ are also constrained:

(A2) $$\sum_{i\in D}p_{i}=P_{D}\;\mathrm{and}\;\;\sum_{j\in \tilde{D}}p_{j}=P_{\tilde{D}}\;,$$

with $P_{D}+P_{\tilde{D}}=1$. Under these special circumstances, constraints on $\tilde{D}$ will not influence $p_{i}$s within D, and vice versa.

To obtain the posterior, maximize S[p,q] subject to these three constraints,

$$\begin{array}{cc} 0 & =\left[\delta S-\lambda \left(\sum_{i\in D}p_{i}-P_{D}\right)+\right.\\ \; & -\left.\tilde{\lambda }\left(\sum_{j\in \tilde{D}}p_{i}-P_{\tilde{D}}\right)+\mu \left(\sum_{j\in \tilde{D}}a_{j}p_{j}-A\right)\right]\;, \end{array}$$

leading to

(A3) $$\begin{array}{cc} \frac{\partial S}{\partial p_{i}} & =\lambda \;\mathrm{for}\;i\in D\;, \end{array}$$

(A4) $$\begin{array}{cc} \frac{\partial S}{\partial p_{j}} & =\tilde{\lambda }+\mu a_{j}\;\mathrm{for}\;j\in \tilde{D}\;. \end{array}$$

Equations (A1)–(A4) are n+3 equations; we must solve for the $p_{i}$s and the three Lagrange multipliers, λ, $\tilde{\lambda }$, and μ. Since $S=S(p_{1}\ldots p_{n},q_{1}\ldots q_{n})$ its derivative

$$\frac{\partial S}{\partial p_{i}}=f_{i}\left(p_{1}\ldots p_{n},q_{1}\ldots q_{n}\right)$$

could, in principle, also depend on all 2n variables. However, this violates the DC1 criterion because any arbitrary change in $a_{j}$ within $\tilde{D}$ would influence the $p_{i}$s within D. The only way that probabilities conditioned on D can be shielded from arbitrary changes in the constraints pertaining to $\tilde{D}$ is that for any i∈D, the function $f_{i}$ depends only on $p_{j}$s with j∈D. Furthermore, this must hold not just for one particular partition of X into domains D and $\tilde{D}$, but it must hold for *all conceivable partitions*, including the partition into atomic propositions. Therefore, $f_{i}$ can depend only on $p_{i}$,

(A5) $$\frac{\partial S}{\partial p_{i}}=f_{i}\left(p_{i},q_{1}\ldots q_{n}\right)\;.$$

The power of the criterion DC1 is not exhausted yet. The information that affects the posterior can enter not just through constraints, but also through the prior. Suppose that the local information about domain $\tilde{D}$ is altered by changing the prior within $\tilde{D}$. Let $q_{j}\to q_{j}+\delta q_{j}$ for $j\in \tilde{D}$. Then (A5) becomes

$$\frac{\partial S}{\partial p_{i}}=f_{i}\left(p_{i},q_{1}\ldots q_{j}+\delta q_{j}\ldots q_{n}\right),$$

which shows that $p_{i}$ with i∈D will be influenced by information about $\tilde{D}$ unless $f_{i}$ with i∈D is independent of all the $q_{j}$s for $j\in \tilde{D}$. Again, this must hold for all possible partitions into D and $\tilde{D}$, and therefore,

$$\frac{\partial S}{\partial p_{i}}=f_{i}\left(p_{i},q_{i}\right)\;\mathrm{for}\;\mathrm{all}\;i\in X\;.$$

The choice of the functions $f_{i}\left(p_{i},q_{i}\right)$ can be restricted further. If we maximize S[p,q], subject to constraints

$$\sum_{i}p_{i}=1\;\mathrm{and}\;\sum_{i}a_{i}p_{i}=A$$

we obtain

$$\frac{\partial S}{\partial p_{i}}=f_{i}\left(p_{i},q_{i}\right)=\lambda +\mu a_{i}\;\mathrm{for}\;\mathrm{all}\;i\in X\;,$$

where λ and μ are Lagrange multipliers. Solving for $p_{i}$ gives a posterior,

$$P_{i}=g_{i}\left(q_{i},\lambda ,\mu ,a_{i}\right)$$

for some functions $g_{i}$. As stated in Section 3.3 we do not assume that the labels *i* themselves carry any particular significance. This means, in particular, that for any proposition labeled *i*, we want the selected posterior $P_{i}$ to depend only on the numbers $q_{i}$, λ, μ, and $a_{i}$. We do not want to have different updating rules for different propositions: two different propositions *i* and $i^{\prime }$ with the same $q_{i}=q_{i^{\prime }}$ and the same $a_{i}=a_{i^{\prime }}$ should be updated to the same posteriors, $P_{i}=P_{i^{\prime }}$. In other words, the functions $g_{i}$ and $f_{i}$ must be independent of *i*. Therefore,

(A6) $$\frac{\partial S}{\partial p_{i}}=f\left(p_{i},q_{i}\right)\;\mathrm{for}\;\mathrm{all}\;i\in X\;.$$

Integrating, one obtains

$$S\left[p,q\right]=\sum_{i}F\left(p_{i},q_{i}\right)+\mathrm{constant}.$$

for some still undetermined function *F*. The constant has no effect on the entropy maximization and can be dropped.

The corresponding expression for a continuous variable *x* is obtained replacing *i* by *x*, and the sum over *i* by an integral over *x* leading to Equation (2),

$$S\left[p,q\right]=\int dx\;F\left(p(x),q(x)\right)\;.$$

## Appendix B. DC2—Independent Subsystems

Here, we show that DC2 leads to Equation (9). Let the microstates of a composite system be labeled by $\left(i_{1},i_{2}\right)\in X=X_{1}\times X_{2}$. We shall consider two special cases.

Case (a)

First, we treat the two subsystems separately. Suppose that for subsystem 1, we have the extremely constraining information that updates $q_{1}\left(i_{1}\right)$ to be $P_{1}\left(i_{1}\right)$, and for subsystem 2 we have no new information at all. For subsystem 1, we maximize $S_{1}\left[p_{1},q_{1}\right]$ subject to the constraint $p_{1}\left(i_{1}\right)=P_{1}\left(i_{1}\right)$ and the selected posterior is, of course, $p_{1}\left(i_{1}\right)=P_{1}\left(i_{1}\right)$. For subsystem 2, we maximize $S_{2}\left[p_{2},q_{2}\right]$ subject only to normalization and there is no update, $P_{2}\left(i_{2}\right)=q_{2}\left(i_{2}\right)$.

When the systems are treated jointly, however, the inference is not nearly as trivial. We want to maximize the entropy of the joint system,

$$S\left[p,q\right]=\sum_{i_{1},i_{2}}F\left(p\left(i_{1},i_{2}\right),q_{1}\left(i_{1}\right)q_{2}\left(i_{2}\right)\right)\;,$$

subject to the constraint on subsystem 1,

$$\sum_{i_{1}}\;p\left(i_{1},i_{2}\right)=P_{1}\left(i_{1}\right)\;.$$

Notice that this is not just one constraint: we have one constraint for each value of $i_{1}$, and each constraint must be supplied with its own Lagrange multiplier, $\lambda_{1}\left(i_{1}\right)$. Then,

$$\delta \left[S-\sum_{i_{1}}\lambda_{1}\left(i_{1}\right)\left(\sum_{i_{2}}\;p\left(i_{1},i_{2}\right)-P_{1}\left(i_{1}\right)\right)\right]=0\;.$$

The independent variations $\delta p(i_{1},i_{2})$ yield the following:

$$f\;\left(p\left(i_{1},i_{2}\right),q_{1}\left(i_{1}\right)q_{2}\left(i_{2}\right)\right)=\lambda_{1}\left(i_{1}\right)\;,$$

where *f* is given in (A6),

$$\frac{\partial S}{\partial p}=\frac{\partial }{\partial p}F\left(p,q_{1}q_{2}\right)=f\left(p,q_{1}q_{2}\right)\;.$$

Next, we impose that the selected posterior is the product $P_{1}\left(i_{1}\right)q_{2}\left(i_{2}\right)$. The function *f* must be such that

$$f\;\left(P_{1}q_{2},q_{1}q_{2}\right)=\lambda_{1}\;.$$

Since the RHS is independent of the argument $i_{2}$, the *f* function must be such that the $i_{2}$-dependence cancels out, and this cancellation must occur for all values of $i_{2}$ and all choices of the prior $q_{2}$. Therefore, we impose that for any value of *x* the function f(p,q) must satisfy

f(px,qx)=f(p,q).

Choosing x=1/q, we obtain

(A7) $$f\left(\frac{p}{q},1\right)=f\left(p,q\right)\;\mathrm{or}\;\frac{\partial F}{\partial p}=f\left(p,q\right)=\phi \left(\frac{p}{q}\right).$$

Thus, the function f(p,q) has been reduced to a function ϕ(p/q) of a single argument.

Case (b)

Next, we consider a situation in which both subsystems are updated by extremely constraining information: when the subsystems are treated separately, $q_{1}\left(i_{1}\right)$ is updated to $P_{1}\left(i_{1}\right)$ and $q_{2}\left(i_{2}\right)$ is updated to $P_{2}\left(i_{2}\right)$. When the systems are treated jointly, we require that the joint prior for the combined system $q_{1}\left(i_{1}\right)q_{2}\left(i_{2}\right)$ be updated to $P_{1}\left(i_{1}\right)P_{2}\left(i_{2}\right)$.

First we treat the subsystems separately. Maximize the entropy of subsystem 1,

$$S\left[p_{1},q_{1}\right]=\sum_{i_{1}}F\;\left(p_{1}\left(i_{1}\right),q_{1}\left(i_{1}\right)\right)\;\mathrm{subject}\;\mathrm{to}\;p_{1}\left(i_{1}\right)=P_{1}\left(i_{1}\right)\;.$$

To each constraint—one constraint for each value of $i_{1}$—we must supply one Lagrange multiplier, $\lambda_{1}\left(i_{1}\right)$. Then, we obtain

$$\delta \left[S-\sum_{i_{1}}\lambda_{1}\left(i_{1}\right)\left(\;p\left(i_{1}\right)-P_{1}\left(i_{1}\right)\right)\right]=0\;.$$

Using Equation (A7),

$$\frac{\partial S}{\partial p_{1}}=\frac{\partial }{\partial p_{1}}F\left(p_{1},q_{1}\right)=\phi \left(\frac{p_{1}}{q_{1}}\right)\;,$$

and, imposing that the selected posterior be $P_{1}\left(i_{1}\right)$, we find that the function ϕ must obey

(A8) $$\phi \left(\frac{P_{1}\left(i_{1}\right)}{q_{1}\left(i_{1}\right)}\right)=\lambda_{1}\left(i_{1}\right)\;.$$

Similarly, for system 2 we find the following:

(A9) $$\phi \left(\frac{P_{2}\left(i_{2}\right)}{q_{2}\left(i_{2}\right)}\right)=\lambda_{2}\left(i_{2}\right)\;.$$

Next, we treat the two subsystems jointly. Maximize the entropy of the joint system as follows:

$$S\left[p,q\right]=\sum_{i_{1},i_{2}}F\;\left(p\left(i_{1},i_{2}\right),q_{1}\left(i_{1}\right)q_{2}\left(i_{2}\right)\right)\;,$$

subject to the following constraints on the joint distribution $p(i_{1},i_{2})$:

$$\sum_{i_{2}}\;p\left(i_{1},i_{2}\right)=P_{1}\left(i_{1}\right)\;\mathrm{and}\;\sum_{i_{1}}\;p\left(i_{1},i_{2}\right)=P_{2}\left(i_{2}\right)\;.$$

Again, there is one constraint for each value of $i_{1}$ and of $i_{2}$ and we introduce Lagrange multipliers, $\eta_{1}\left(i_{1}\right)$ or $\eta_{2}\left(i_{2}\right)$. Then,

$$\delta \left[S-\sum_{i_{1}}\eta_{1}\left(i_{1}\right)\left(\sum_{i_{2}}\;p\left(i_{1},i_{2}\right)-P_{1}\left(i_{1}\right)\right)-\left\{1↔2\right\}\right]=0,$$

where {1↔2} indicates a third term, similar to the second, with 1 and 2 interchanged. The independent variations $\delta p(i_{1},i_{2})$ yield

$$\phi \left(\frac{p(i_{1},i_{2})}{q_{1}\left(i_{1}\right)q_{2}\left(i_{2}\right)}\right)=\eta_{1}\left(i_{1}\right)+\eta_{2}\left(i_{2}\right)\;,$$

and we impose that the selected posterior be the product $P_{1}\left(i_{1}\right)P_{2}\left(i_{2}\right)$. Therefore, the function ϕ must be such that

$$\phi \left(\frac{P_{1}P_{2}}{q_{1}q_{2}}\right)=\eta_{1}+\eta_{2}\;.$$

To solve this equation, we take the exponential of both sides, let ξ=expϕ, and rewrite as

(A10) $$\xi \left(\frac{P_{1}P_{2}}{q_{1}q_{2}}\right)e^{-\eta_{2}\left(i_{2}\right)}=e^{\eta_{1}\left(i_{1}\right)}\;.$$

This shows that for any value of $i_{1}$, the dependences of the LHS on $i_{2}$ through $P_{2}/q_{2}$ and $\eta_{2}$ must cancel each other out. In particular, if for some subset of $i_{2}$s, the subsystem 2 is updated so that $P_{2}=q_{2}$, which amounts to no update at all, the $i_{2}$ dependence on the left is eliminated but the $i_{1}$ dependence remains unaffected,

$$\xi \left(\frac{P_{1}}{q_{1}}\right)e^{-\eta_{2}^{\prime }}=e^{\eta_{1}\left(i_{1}\right)}\;.$$

where $\eta_{2}^{\prime }$ is some constant independent of $i_{2}$. A similar argument with {1↔2} yields

$$\xi \left(\frac{P_{2}}{q_{2}}\right)e^{-\eta_{1}^{\prime }}=e^{\eta_{2}\left(i_{2}\right)}\;,\;$$

where $\eta_{1}^{\prime }$ is a constant. Taking the exponential of (A8) and (A9) leads to the following:

$$\xi \left(\frac{P_{1}}{q_{1}}\right)e^{-\eta_{2}^{\prime }}=e^{\lambda_{1}-\eta_{2}^{\prime }}=e^{\eta_{1}}\;\mathrm{and}\;\xi \left(\frac{P_{2}}{q_{2}}\right)e^{-\eta_{1}^{\prime }}=e^{\lambda_{2}-\eta_{1}^{\prime }}=e^{\eta_{2}}\;.$$

Substituting back into (A10), we obtain

$$\xi \left(\frac{P_{1}P_{2}}{q_{1}q_{2}}\right)=\xi \left(\frac{P_{1}}{q_{1}}\right)\xi \left(\frac{P_{2}}{q_{2}}\right)\;,$$

where a constant factor $e^{-(\eta_{1}^{\prime }+\eta_{2}^{\prime })}$ is absorbed into a new function ξ. The general solution of this functional equation is a power,

$$\xi \left(xy\right)=\xi \left(x\right)\xi \left(y\right)⟹\xi \left(x\right)=x^{a}\;,$$

so that

ϕ(x)=alogx+b,

where *a* and *b* are constants. Finally, integrate (A7),

$$\frac{\partial F}{\partial p}=\phi \left(\frac{p}{q}\right)=a\log\frac{p}{q}+b\;,$$

to obtain

$$F\left[p,q\right]=ap\log\frac{p}{q}+b^{\prime }p+c$$

where $b^{\prime }$ and *c* are constants.

At this point, the entropy takes the general form

$$S\left[p,q\right]=\sum_{i}\left(ap_{i}\log\frac{p_{i}}{q_{i}}+b^{\prime }p_{i}+c\right).$$

The additive constant *c* may be dropped: it contributes a term that does not depend on the probabilities and has no effect on the ranking scheme. Furthermore, since S[p,q] will be maximized subject to constraints that include normalization, the $b^{\prime }$ term has no effect on the selected distribution and can also be dropped. Finally, the multiplicative constant *a* has no effect on the overall ranking, except in the trivial sense that inverting the sign of *a* will transform the maximization problem to a minimization problem or vice versa. We can, therefore, set a=−1 so that maximum *S* corresponds to maximum preference, which gives us Equation (9) and concludes our derivation.
